# Student supervision by trainee doctors in GP teaching practices: Win-win situation or additional burden? An interview study on current practices and acceptance

**DOI:** 10.3205/zma001701

**Published:** 2024-09-16

**Authors:** Sabine Gehrke-Beck, Ulrike Sonntag, Tomke Schubert, Mariyan Madzharov, Bert Huenges

**Affiliations:** 1 Charité – University Hospital Berlin, Institute of General Practice, Berlin, Germany; 2 Ruhr University Bochum, Department for General Practice, Bochum, Germany

**Keywords:** general practice, junior physicians, undergraduate medical education, vocational training, teaching methods

## Abstract

**Objective:**

Teaching by trainee doctors is also established practice in general practice in English-speaking countries. This study examines the involvement of trainee doctors in the supervision of students in German general practices and the acceptance of trainee doctors as teachers from the perspective of physicians with a license for post-graduate training (PLT) and the trainee doctors themselves.

**Methodology:**

Semi-structured qualitative interviews were conducted with 9 PLTs and 9 trainees. The interview guide was developed based on the Theoretical Framework of Acceptance. Interviews were recorded, transcribed and evaluated using Kuckartz’s qualitative analysis.

**Results:**

Trainee doctors are involved in student supervision in GP teaching practices to varying degrees and often in unstructured ways. Supervision by trainees is considered advantageous as they are closer in terms of hierarchy, possess more up-to-date knowledge and are less far ahead in terms of knowledge and function as role models. However, professional uncertainty or revealing knowledge gaps to patients and students is experienced as difficult by some trainees. Competing for time with patient care is seen as a challenge. Better time planning and didactic preparation could avoid pressure in this area. Teaching is seen as part of the GP profession, especially by trainee doctors. However, a potential obligation to teach is seen as more of a hindrance to encouraging the next generation of doctors by both trainee doctors and PLTs.

**Conclusion:**

The inclusion of trainee doctors in student teaching is frequently practiced by those surveyed, which suggests a high level of acceptance but is not consistently implemented. Structured organization of teaching in real life, didactic qualifications and offering credits for teaching activities might further improve inclusion and acceptance.

## Zusammenfassung

**Zielsetzung:**

Lehre durch Ärzt*innen in Weiterbildung (ÄiW) ist in angelsächsischen Ländern auch in der Allgemeinmedizin etabliert. Diese Studie untersucht die Einbindung von ÄiW in die Betreuung von Studierenden in deutschen Hausarztpraxen und die Akzeptanz von ÄiW als Lehrende aus der Sicht von Weiterbildungsbefugten (WBB) und ÄiW selbst.

**Methodik:**

Es wurden semistrukturierte qualitative Interviews mit 9 WBB und 9 ÄiW durchgeführt. Der Interviewleitfaden wurde ausgehend vom Theoretical Framework of Acceptance entwickelt. Interviews wurden aufgezeichnet, transkribiert und mit der qualitativen Analyse nach Kuckartz ausgewertet.

**Ergebnisse:**

ÄiW werden in den befragten hausärztlichen Lehrpraxen in heterogenem Umfang und häufig unstrukturiert in die Studierendenbetreuung eingebunden. Die Betreuung durch ÄiW wird durch die geringere Hierarchie, aktuelleres Wissen und weniger Wissensvorsprung und ihre Vorbildfunktion als vorteilhaft erachtet. Fachliche Unsicherheit bzw. Offenlegen von Wissenslücken vor Patient*innen und Studierenden wird jedoch von einigen ÄiW als schwierig erlebt. Die zeitliche Konkurrenz zur Patient*innenversorgung wird als Herausforderung benannt. Hier könnte eine bessere zeitliche Planung und didaktische Vorbereitung Belastungen vermeiden. Lehre wird vor allem von ÄiW als Teil des hausärztlichen Berufsbilds betrachtet. Verpflichtungen zur Lehrtätigkeit werden jedoch sowohl von ÄIW als auch von WBB eher als hinderlich für die Nachwuchsförderung eingeschätzt.

**Schlussfolgerung:**

Die Einbindung von ÄiW im Studierendenunterricht wird von den Befragten häufig praktiziert, was auf eine hohe Akzeptanz schließen lässt, aber sehr heterogen umgesetzt. Strukturierte Lehrorganisation in der Praxis, didaktische Qualifizierung und Anerkennung der Lehrtätigkeit könnten Einbindung und Akzeptanz weiter verbessern.

## 1. Introduction

Peer teaching and near-peer teaching are increasingly being used as teaching methods in medical studies because they offer a variety of advantages [32], [39], [42]. The involvement of trainee doctors as near-peer teachers has been well-researched and shows both didactic advantages and offers relief for specialist doctors [7], [18], [26]. Students estimate that about a third of their knowledge is imparted by trainee doctors [5]. The Regulation for Postgraduate Medical Education in Germany currently does not call for didactic skills to be acquired during postgraduate medical education and it is unclear to what extent trainee doctors are involved in student supervision. 

Teaching practices are an effective location for learning with 1:1 student supervision but with a correspondingly effort in terms of human resources. Involving trainee doctors to supervise students may be particularly helpful here. In English-speaking countries, such postgraduate training in General Practice are known as “residents as teachers” programs [1], [27], “near-peer teaching” [17], [40] or “vertical teaching” [2], [3], [9]. Studies from these countries show that a large proportion of trainee doctors supervise students and view this positively [15], [29], [36], [41], [43], as the trainees see this as an opportunity to improve their own competences and as help in patient care [20], [21], [36]. Specialist doctors providing training acknowledge that this relieves their own time pressures but are to an extent skeptical about transferring responsibility for teaching [20], [28], [33], [36]. It is still unclear whether these findings can be transferred to the situation in Germany, as there are significant differences both in under- and postgraduate medical education and the organization of primary care. 

This study aims to determine what the involvement of trainee doctors is in supervising students in GP practices and how accepted the concept of near-peer teaching has become in everyday life from the perspective of trainee doctors and physicians with a license for post-graduate training (PLTs).

## 2. Methods

### 2.1. Research approach

In the critical realism approach [12], [22], [38], behavior and attitudes are seen to be significantly shaped by social framework conditions and interactions [22]. A qualitative research approach is suitable for comprehensively collecting aspects that influence actions and attitudes. A qualitative survey was therefore chosen in order to understand the processes of under- and postgraduate medical education in practices from the perspective of those involved. Trainee doctors and PLTs were interviewed as experts in terms of routine under- and postgraduate medical education in GP practices using semi-structured interviews. 

### 2.2. Interview guide

The Theoretical Framework of Acceptance [35] was used to develop the guide. The model is used in implementation research to examine the acceptance of interventions in daily routines. It distinguishes 7 central aspects: the affective attitude, the burden, the ethicality, and the intervention coherence. Opportunity costs, perceived effectiveness and self-efficacy. Questions and narrative stimuli were developed for the guide and discussed in the qualitative research workshop at the Institute of General Practice at the Charité (see attachment 1 ).

### 2.3. Sampling

Recruitment took place in the teaching practice network locally in Berlin and nationwide via the Society of University Teaching Staff in general medicine and the Competence Centers for Postgraduate Medical Education in general medicine. Teaching coordinators or heads of the competence centers asked physicians with a license for post-graduate training in practices who regularly supervise students whether they would like to take part in the study. To ensure maximum variation sampling, participants with varying professional and teaching experience and from different practice settings were recruited. Of the 17 PLTs contacted, nine took part (four did not respond, four declined to take part due to lack of time). The PLTs made contact to those trainee doctors in their practices who had agreed to participate. All nine trainees contacted took part (from a total of 7 practices, it was not possible to get in touch with trainees at two practices and in two practices two trainees agreed to participate). No relevant new aspects were added in the last four interviews. The participants gave their written consent to the interviews, the audio recording and data processing. 

### 2.4. Data collection

The interviews were conducted by the first author (SGB) from October to December 2023. The interviewer works both at the university and in a General Practice and is therefore familiar with both the theory and implementation of teaching in practices. Individual respondents were known to the interviewer from previous professional contacts. The interviews were planned using Zoom video-conferencing software with digital sound recording; if participants requested this, interviews were also conducted in person or by telephone. 

### 2.5. Analysis

The interviews were transcribed according to Kuckartz ([23], p.200) and evaluated using structuring qualitative content analysis with the help of MAXQDA software [23]. After initial text exploration and case summaries ([23], p.118ff), main categories were developed deductively ([23], p.71-72). Four interviews were counter-coded by two team members (MM, TS), and different codings were discussed. Subcategories were added inductively ([23], p.138ff) and discussed and agreed upon with two team members (US, TS). An initial summary of the results was sent to the interview participants for member checking and for feedback on the results [13]. 

## 3. Results

### 3.1. Participants 

18 interviews were conducted (six in person, 11 online, one by phone) with an average duration of 24 minutes (16-35 minutes). The interviews with trainee doctors were on average slightly longer (26 vs. 22 minutes). Information about the participants is shown in table 1 (Tab. 1).

### 3.2. System of categories

To begin with, the reports on the role of trainees in routine patient consultations and in supervising students are presented. The acceptance of student supervision by trainees in routine practice operations is then reported in a structured manner according to the Theoretical Framework of Acceptance. The System of Categories developed can be found in attachment 2 . The aspects of the theoretical framework of acceptance are presented in the order of relevance they had in the interviews.

### 3.3. Role of trainees in routine patient consultations and in student supervision

In the interviews, the role of trainees in patient consultations is predominantly presented as comparable to and equal to that of specialist doctors. After on-boarding, a process which differs in structure from practice to practice, the trainees treat all patient groups largely independently. Longer appointments are usually scheduled for trainee doctors so that they can consult and read up without time pressure. 

There are different reports on the role of trainees in supporting students. In some practices, trainee doctors do not supervise students at all, in others they do so occasionally or depending on the situation, for example when PLTs are busy with other tasks. Some PLTs employ trainee doctors in a role comparable to that of other specialists, for example by having them supervise students on a rotating basis. Some report that students are predominantly supervised by trainee doctors or that they are the main contact person. In terms of organization, student supervision by trainee doctors often occurs spontaneously and in a rather unplanned fashion; supervision is rarely planned and agreed in advance. The main initiative comes from the PLT. The willingness to supervise a student is not always queried but the trainee doctors surveyed were happy to provide student supervision. Trainee doctors take students into their consulting rooms, to home visits and examinations, show them interesting cases and supervise advanced students examining patients themselves. 

### 3.4. Self-efficacy – are trainee doctors capable of supervising students? 

#### 3.4.1 Gaps in knowledge and uncertainty

Dealing with gaps in knowledge and uncertainty is mentioned by many trainee doctors and PLTs. Personal professional uncertainty is not generally seen as a problem in student supervision. Trainee doctors and PLTs emphasize that asking questions or looking something up in the presence of students is not an issue.

*“They get a very concrete idea of how, being new to the profession, I cope with that and they also learn from my difficulties, learning how to swim, or from the questions I have or they just get to know my work is for me and how I’m learning to swim myself, so to speak. So that’s something that I am happy to share and which does not put any additional pressure on me, I would say, yes, exactly.” (Trainee 5)*

The majority of trainee doctors and PLTs see a clear advantage in terms of competence for trainees through practical experience in the profession and thus the opportunity to pass on practical knowledge to students. 

Some trainee doctors experience the remaining uncertainty as a difficulty in supervising students in a GP practice, as it requires a real breadth of knowledge. Several PLTs see problems when trainee doctors with no prior clinical experience supervise students, having just been on-boarded themselves and not yet fully familiar with the work and, in individual cases, when trainee doctors themselves have insufficient knowledge. 

#### 3.4.2. Didactic skills

Many trainee doctors and PLTs consider didactic qualifications to be helpful but only a few see them as critical and most trainee doctors supervise students without specific didactic preparation. Some see it as useful and sufficient to learn how to supervise students from role models at work. Internal didactic considerations on how students can be supervised well are mentioned as another way to gain more confidence. 

*“I have never been given special teaching materials or was told: Just take that patient, that’s a great teaching example, that’s something that could be done” (Trainee 6)*

### 3.5. Effectiveness – benefits for students and trainee doctors

Trainee doctors and PLTs agreed that students value and welcome supervision by trainee doctors. It is seen as positive that students feel more comfortable with younger colleagues and can ask more open questions. In addition, PLTs in particular mention the advantage that trainee doctors bring other knowledge and skills with them, for instance more up-to-date knowledge from their studies and clinical training, knowledge of sonography and information on postgraduate medical education. Because they are only a few years ahead of the students in their careers, trainee doctors are well-suited as role models for someone starting out on their career and can demonstrate how to deal with uncertainty and patients. Another advantage is seen in the fact that by involving trainee doctors, there are more teachers present to supervise and provide input. It is suggested that ideally both trainee doctors and specialist doctors should be employed as teachers, playing to the strength of each group. 

*“Well, I think, in terms of expertise, in our current setup, you can learn more from (physicians with a license for post-graduate training) and maybe they a few things from me, but you can learn a lot more from a trainee doctor, how do I feel in the beginning, in dealing with uncertainties, sometimes - let’s say - incompetence, so, well, that’s something too.” (Trainee 7)*

Trainee doctors and PLTs also find teaching useful for their own learning. Specifically, it is reported that questions from students reveal gaps in their own knowledge and encourage them to prepare and read up on things. Both groups also emphasize that they also learn from students who bring current knowledge from their studies. 

*“Let’s take the intern, who is more versed in theory than I am in many things I’m sure, and I suspect the same is true for the trainee doctor, that they use the potential that the students have, I suspect.” (PLT 7)*

### 3.6. Emotional attitude – enjoyment of teaching

Most interviewees report that trainee doctors have a positive attitude towards supervising students. The opportunity to pass something on to someone and to support their personal development, as well as being with young people and working together when seeing a patient all contribute to the enjoyment of teaching. In addition, it is mentioned – more frequently by PLTs – that trainee doctors gain pride and self-confidence from being more knowledgeable than the students. 

### 3.7. Opportunity costs – planning teaching

Most trainee doctors and PLTs mention the extra time required for student supervision and didactic training. Trainee doctors in particular would like time to be actively scheduled for this. Advance planning of supervision with backup activities for students, for example supervision by medical specialists or independent practice, is seen as helpful. PLTs and some trainee doctors state that trainee doctors are generally allocated a more generous time allowance at work and that teaching is therefore easier for them to integrate. Many PLTs report that it relieves time pressures on themselves when trainee doctors are involved in student supervision. Several trainee doctors and individual PLTs experience benefits for patients through student supervision, having a second set of eyes and ears and with students have more time for detailed examinations and taking patient histories. 

*“Personally, I didn’t have the feeling that it took more time to speak to the student again afterwards but I think it’s actually positive for the quality of supervision when they pick up on something that you yourself may not have noticed at the time. It’s definitely good when something is corrected, even if by a student.” (Trainee 1)*

### 3.8. Stress – meeting expectations

The stress caused by supervising students is mentioned by individual trainee doctors and PLTs. The pressure having to meet the needs of students and patients at the same time and potentially having to disclose knowledge gaps in front of students or patients were experienced as stressful. 

*“But above all, you tell yourself, there’s a bit of a hurdle to overcome, an obligation, a three-way obligation, that I want to meet the needs of both student and patient.” (Trainee 3)*

### 3.9. Ethics – student presence in consultations

Ethical challenges are only mentioned in individual interviews with trainee doctors. These trainee doctors asked themselves whether patients were always ok with a student being present in the consultation. There was still some uncertainty as to whether, for example, patients with psychiatric problems, might have felt more comfortable without the student, but did not want to refuse. 

### 3.10. Coherence – is teaching part of the job description of a GP?

#### 3.10.1. Teaching as postgraduate medical education content

In the interviews, personal learning experiences were often mentioned in which near-peer teaching was experienced as useful and which shape the attitude towards teaching. For most respondents, taking on teaching tasks during postgraduate medical education is a coherent concept that should be encouraged and prepares for later teaching or continuing education tasks. Mandatory introduction of teaching skills in the postgraduate education catalog is seen critically, less so in terms of content but mostly from an organizational perspective. As additional courses are already required in postgraduate medical education, e.g. a course on psychosomatics, introducing yet another mandatory skill is seen as a barrier. In addition, only some of the postgraduate education practices offer students supervision and acquiring this competence could become a bottleneck in postgraduate medical education were it to be mandatory. From the respondents’ point of view, it would be better to offer didactic training to make teaching opportunities in postgraduate medical education more widely known. It is emphasized that PLTs should also be encouraged to include trainee doctors in student supervision, for example in train-the-trainer seminars. Best practice examples of effective teaching organization could be published and presented. 

#### 3.10.2. Teaching as part of the professional profile

The respondents mostly see teaching as part of their professional profile as a GP. Teaching is seen on the one hand as an aspect of one’s own lifelong learning, and on the other hand as a necessary justification and explanation of one’s own evidence-based actions. Another aspect mentioned is encouraging the next generation of medical professionals. 

*“Actually, I think it makes sense, yes. I just think that you always learn a lot, especially when you explain, teach others how and why you do something, so you definitely question what you are doing again.” (Trainee 6)*

In contrast, some see teaching as something suited to certain people or as an additional task for particularly interested GPs. Even those who considered teaching to be part of a GP’s job in principle stressed that it may not be possible to oblige everyone to do it, as a lack of interest or suitability can lead to poor teaching and dissatisfaction among teachers and students.

Another way of promoting teaching in GP practices would be to provide financial compensation for offering student supervision or credit for CME training or as a criterion for obtaining a longer training authorization for the practice.

## 4. Discussion

### 4.1. Summary of the results

The respondents see clear strengths in involving trainee doctors in student supervision. There are positive effects for students, the trainee doctors’ own acquisition of skills and for patient care. Professional uncertainties of trainee doctors are seen more as an opportunity to show students how to deal with knowledge gaps. A lack of didactic preparation is identified as a weakness.

The time required for student supervision is experienced as a challenge. Scheduling students in consultation hours with appropriate time slots and independent learning activities or supported by other team members could make routine teaching easier.

Teaching is predominantly seen as a relevant aspect of a GP’s work and the involvement of trainee doctors in teaching tasks as an opportunity to strengthen teaching in GP practice. Mandatory requirements are viewed critically and support through best practice examples and training as well as financial and non-financial recognition are seen as having more potential. 

### 4.2. Comparison with literature

Comparable to international surveys [10], [15], [29], [36], [43], respondents have a predominantly positive attitude towards student supervision despite the different framework conditions in German GP practices. The low-hierarchy learning atmosphere with trainee doctors, similarities in knowledge perspectives and the role model function are also cited as advantages in other studies in clinical and practice settings [14], [17], [18], [25], [26]. Trainee doctors thus create the conditions for trusted learning in the sense of experiential learning to a high degree [11]. Skepticism on the part of the PLTs is described internationally [14], [33], [36] but in the case of the PLTs surveyed here, this skepticism relates more to justified individual cases such as incomplete on-boarding, newcomers to the profession or individuals with clear knowledge deficits. 

Didactic training courses were requested in the interviews and have already been conceptually developed and evaluated internationally for this target group [31] and [6], [9], [30]. This leads to trainee doctors feeling more competent and teaching more but also complaints about the extra time required.

Workload due to patient care and time pressure are described internationally as a problem by trainee doctors; likewise, PLTs are concerned about the trainee doctors being overworked [8], [16], [21], [22]. In Germany, practices are currently not allowed to bill for additional patients when employing trainee doctors, so that longer appointments can be scheduled in patient care training, something mentioned by many respondents. This allows trainee doctors to spend more time with students and relieves the burden on PLTs.

### 4.3. Strengths and weaknesses

Through the qualitative survey from the two perspectives – that of trainee doctors and PLTs – the study is able to provide a comprehensive picture of how trainee doctors are integrated into student supervision in everyday practice and the circumstances and attitudes underlying this. The theoretical framework made it possible to gather a comprehensive picture of possible relevant aspects and present them in a structured manner.

Among the participants, there were more trainee doctors in the later stages of their postgraduate training and more PLTs with many years of professional and teaching experience, as these groups predominate in everyday German health care but the perspective of those with less experience was explicitly included. Contrary to the current distribution of practices, larger practices are more common in the sample than individual practices. This is helpful for considering future acceptance, as junior doctors prefer practices with colleagues or employment options [19]. 

Socially desirable answers cannot be ruled out, especially since the interviewer knows some of the respondents through her professional activities. Stress and uncertainty were nevertheless addressed in the interviews. During recruitment, an attempt was made to include practices with little teaching experience but it is likely that those who are particularly interested and doctors who are particularly committed to teaching are more willing to take part in interviews. 

As the study was conducted without financial resources, the interviews were largely coded by the main author alone. The study design and interview guide as well as the results were discussed in a research workshop and people with different backgrounds were involved in the coding (a student, a psychologist working in the field of continuing education and a teaching administrator).

The approach chosen was purely qualitative; feedback bias cannot be ruled out. To estimate the extent of acceptance of using of trainee doctors in student teaching in General Practice and the frequency of different work practices, the results should be supported by quantitative data from representative samples. 

## 5. Conclusions: Strategies for teaching in postgraduate medical education

Peer teaching is a concept that is not only suitable for (general) medical under- or postgraduate medical education [24], [34]. Near-peer teaching of students by trainee doctors can have positive effects for both learners and teachers, but which to date are not used much systematically in Germany. 

Strategies can be developed from the strengths and weaknesses of current practice to enable more trainee doctors to gain experience in student supervision and to strengthen GP practices as a places of learning in under- and postgraduate medical education. The strengths of student supervision by trainee doctors can be better utilized through a more structured implementation of student supervision. Clear scheduling, agreements and organizational considerations regarding possible learning activities of students in practice can reduce stress. Since the integration of student supervision would largely be the responsibility of PLTs, structured organization of joint responsibility student supervision should be addressed at this level, for example by presenting best practice examples at train-the-trainer events.

While knowledge gaps are not seen as a barrier to student supervision, a lack of didactic preparation is a complaint which is occasionally raised. Didactic training courses can address the challenges identified in student supervision – especially dealing with one’s own professional uncertainty – for trainee doctors. Last but not least, asking trainee doctors to teach can help them to address their own knowledge gaps more intensively and thereby result in learning successes in their own professional development [4], [20], [21].

Obligations to teach are viewed critically, although many respondents agree that these tasks are part of a GP’s job description. Instead, recognition of student supervision as CPD or as an infrastructure feature for longer training authorization is suggested.

## Notes

### Abbreviations

- Trainees: Trainee doctors
- PLT: Physician with a license for post-graduate training

### Ethics vote and data protection

This study was approved by the Charité Ethics Committee – University Hospital Berlin (EA_177_23 dated 30 August 2023). A data protection concept was created for the study with the support of the data protection team of the Clinical Trial Office (CTO) of the Charité – University Hospital Berlin and reviewed by them. 

### Authors’ ORCIDs

- Sabine Gehrke-Beck: [0000-0002-6221-2813]
- Ulrike Sonntag: [0000-0001-9576-2734]
- Bert Huenges: [0009-0009-3445-0871]

## Acknowledgements

Many thanks go to all interview participants who took the time to complete the survey during the busy bug season, to Christien Radecki for her support in transcribing the interviews; and to the participants of the research workshop at the Institute of General Practice for the opportunity to discuss and for suggestions on conception and evaluation. 

## Competing interests

The authors declare that they have no competing interests. 

## Supplementary Material

Interview guide

Category system and example codes

## Figures and Tables

**Table 1:**
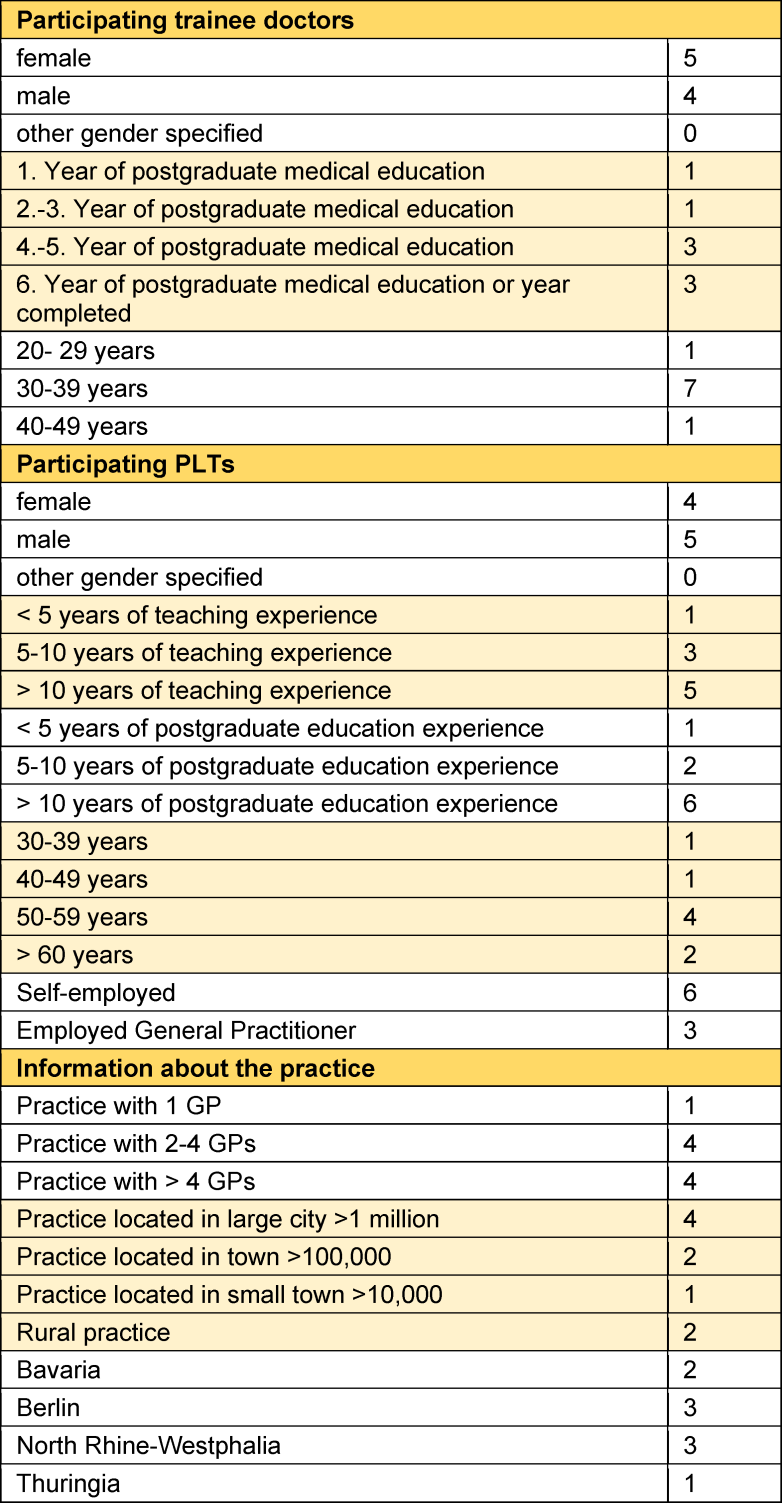
Information about interview participants and practices

## References

[1] Al Achkar M, Hanauer M, Morrison EH, Davies MK, Oh RC (2017). Changing trends in residents-as-teachers across graduate medical education. Adv Med Educ Pract.

[2] Alberti H, Rosenthal J, Kirtchuk L, Thampy H, Harrison M (2019). Near peer teaching in general practice: option or expectation?. Educ Prim Care.

[3] Anderson K, Thomson J (2009). Vertical integration - Reducing the load on GP teachers. Aust Fam Physician.

[4] Avonts M, Michels NR, Bombeke K, Hens N, Coenen S, Vanderveken OM, De Winter BY (2022). Does peer teaching improve academic results and competencies during medical school? A mixed methods study. BMC Med Educ.

[5] Bing-You RG, Sproul MS (1992). Medical students' perceptions of themselves and residents as teachers. Med Teach.

[6] Brand MW, Ekambaram V, Tucker P, Aggarwal R (2013). Residents as teachers: psychiatry and family medicine residents' self-assessment of teaching knowledge, skills, and attitudes. Acad Psychiatry.

[7] Busari JO, Scherpbier AJ (2004). Why residents should teach: a literature review. J Postgrad Med.

[8] de Villiers MR, Cilliers FJ, Coetzee F, Herman N, van Heusden M, von Pressentin KB (2014;22). Equipping family physician trainees as teachers: a qualitative evaluation of a twelve-week module on teaching and learning. BMC Med Educ.

[9] Dick ML, King DB, Mitchell GK, Kelly GD, Buckley JF, Garside SJ (2007). Vertical integration in teaching and learning (VITAL): an approach to medical education in general practice. Med J Aust.

[10] Dodd J, Vickery A, van Osch H, Emery J (2009). General practice registrar teaching roles - is there a need for shared understanding?. Aust Fam Physician.

[11] Dornan T, Conn R, Monaghan H, Kearney G, Gillespie H, Bennett D (2019). Experience Based Learning (ExBL): Clinical teaching for the twenty-first century. Med Teach.

[12] Fletcher AJ (2017). Applying Critical Realism in Qualitative Research: Methodology Meets Method. Int J of Soc Res Meth.

[13] Flick U, Baur N, Blasius J (2022). Gütekriterien qualitativer Sozialforschung.

[14] Gray D, Cozar O, Lefroy J (2017). Medical students' perceptions of bedside teaching. Clin Teach.

[15] Halestrap P, Leeder D (2011). GP registrars as teachers: a survey of their level of involvement and training. Educ Prim Care.

[16] Hayden C, Raidan J, Rees J, Oswal A (2021). Understanding junior doctors' experiences of teaching on the acute take: a qualitative study. BMC Med Educ.

[17] Jones M, Kirtchuk L, Rosenthal J (2020). GP registrars teaching medical students- an untapped resource?. Educ Prim Care.

[18] Karani R, Fromme HB, Cayea D, Muller D, Schwartz A, Harris IB (2014). How medical students learn from residents in the workplace: a qualitative study. Acad Med.

[19] Kassenärztliche Bundesvereinigung (2022). Berufsmonitoring Medizinstudierende.

[20] Kirby J, Rushforth B, Nagel C, Pearson D (2014). Should GP specialty trainees teach? Contrasting views from GP specialty trainees and their trainers. Educ Prim Care.

[21] Kleinitz A, Campbell D, Walters L (2014). General practice registrar perceptions on training medical students. Aust Fam Physician.

[22] Koopmans E, Schiller DC (2022). Understanding Causation in Healthcare: An Introduction to Critical Realism. Qual Health Res.

[23] Kuckartz U, Rädiker S (2022). Qualitative Inhaltsanalyse. Methoden, Praxis, Computerunterstützung.

[24] Ledig T, Eicher C, Szecsenyi J (2014). AaLplus– ein Anamnese- und Untersuchungskurs für den vorklinischen Studienabschnitt. Z Allg Med.

[25] Lockspeiser TM, O'Sullivan P, Teherani A, Muller J (2008). Understanding the experience of being taught by peers: the value of social and cognitive congruence. Adv Health Sci Educ Theory Pract.

[26] Montacute T, Chan Teng V, Chen Yu G, Schillinger E, Lin S (2016). Qualities of Resident Teachers Valued by Medical Students. Fam Med.

[27] Morrison EH, Friedland JA, Boker J, Rucker L, Hollingshead J, Murata P (2001). Residents-as-teachers training in US residency programs and offices of graduate medical education. Acad Med.

[28] Nagel C, Kirby J, Rushforth B, Pearson D (2011). Foundation Programme doctors as teachers. Clin Teach.

[29] Ng VK, Burke CA, Narula A (2013). Residents as teachers: survey of Canadian family medicine residents. Can Fam Physician.

[30] Qureshi ZU, Gibson KR, Ross MT, Maxwell S (2013). Perceived tutor benefits of teaching near peers: insights from two near peer teaching programmes in South East Scotland. Scott Med J.

[31] Ramani S, Mann K, Taylor D, Thampy H (2016). Residents as teachers: Near peer learning in clinical work settings: AMEE Guide No. 106. Med Teach.

[32] Rees EL, Quinn PJ, Davies B, Fotheringham V (2016). How does peer teaching compare to faculty teaching? A systematic review and meta-analysis. Med Teach.

[33] Rushforth B, Kirby J, Pearson D (2010). General practice registrars as teachers: a review of the literature. Educ Prim Care.

[34] Schuetz E, Obirei B, Salat D, Scholz J, Hann D, Dethleffsen K (2017). A large-scale peer teaching programme – acceptance and benefit. Z Evid Fortbild Qual Gesundhwes.

[35] Sekhon M, Cartwright M, Francis JJ (2017). Acceptability of healthcare interventions: an overview of reviews and development of a theoretical framework. BMC Health Serv Res.

[36] Silberberg P, Ahern C, van de Mortel T (2013). ‘Learners as teachers’ in general practice: stakeholders’ views of the benefits and issues. Educ Prim Care.

[37] Stocks NP, Frank O, Linn AM, Anderson K, Meertens S (2011). Vertical integration of teaching in Australian general practice--a survey of regional training providers. Med J Aust.

[38] Sturgiss EA, Clark AM (2020). Using critical realism in primary care research: an overview of methods. Fam Pract.

[39] Ten Cate O, Durning S (2007). Peer teaching in medical education: twelve reasons to move from theory to practice. Med Teach.

[40] Thampy H, Kirtchuk L, Rosenthal J (2019). Near peer teaching in general practice. Br J Gen Pract.

[41] van de Mortel TF, Silberberg PL, Ahern CM, Pit SW (2016). Supporting near-peer teaching in general practice: a national survey. BMC Med Educ.

[42] Vogel B, McMillan A, Dethleffsen K, Noller J, Beitz-Radzio C, Kugelmann D, Sontheimer S, Westerholz S (2019). Peer-Assisted Learning – mehr als eine Methode.

[43] Williams B, Amiel C (2012). General practice registrars as teachers: a questionnaire-based evaluation. JRSM Short Rep.

