# Supplementary material for: Student supervision by trainee doctors in GP teaching practices: Win-win situation or additional burden? An interview study on current practices and acceptance
Source: GMS J Med Educ. 2024 Sep 16;41(4):Doc46. doi: 10.3205/zma001701 (PMC11474648; doi:10.3205/zma001701)
Supplement: Interview guide [file JME-41-46-s-001.pdf]

Attachment 1: Interview guide

**Concept of the theoretical framework**	**Questions for trainees**	**Questions for PLT**
Sociodemographic information<br>Professional experience<br>Practice information<br>Practice type, services, practice customers	Short questionnaire	Short questionnaire
Practice routine - consultations<br>Practice routine - teaching and specialization	What is your role in daily practice routine as part of the team?<br>What is it like when students are in the practice?<br>*to be clarified/ask if necessary:*<br>*How often or regularly?*<br>*On whose initiative? (How did this come about?)*<br>What experiences have you had/collected in other postgraduate education phases previously?<br>*Have you supervised/taught students in postgraduate education to date?*<br>*Follow-on questions: in an in-patient setting? Other practices? Experienced differently?*	What role does the trainee have in the daily practice routine as part of the team? What is it like when students are in the practice?<br>*to be clarified/ask if necessary:*<br>*what involvement in supervision? What tasks? How often or regularly?*<br>*On whose initiative was the involvement? (How did this come about?)*<br>Have you previously involved other trainees in student supervision?<br>*Follow-on questions: how regularly? Depending on what? (Trainee/duration of postgraduate training/practice factors?)*
Advantages/positive effects<br><br><u>Theoretical Framework of Acceptance (TFA):</u><br>Affective attitude<br>Effectiveness<br>Opportunity cost	What consequences/effects do you see when you supervise students (advantages or disadvantages)?<br>*Follow-on questions:*<br>*For students?*<br>*For the practice/the PLTs?*<br>*For patients?*<br>*For yourself?*	Why do you involve trainees in student supervision? / What advantages/effects do you see when trainees supervise students?<br>*Follow-on questions:*<br>*For students?*<br>*For trainees?*<br>*For patients/practice?*<br>*For yourself?*
Competence<br><br><u>TFA:</u><br>Self-Efficacy<br>(Ethicality?)	How well prepared and able do you feel to supervise students?<br>*Follow-on questions: prepared by what? Support? Needs? Previous teaching experience?*	How well are trainees prepared/able to supervise students?<br>*Follow-on questions:*<br>*Depending on what? (Previous experience, duration of postgraduate education/personality? Area of activity?)*

Concept of the theoretical framework	Questions for Trainees	Questions for PLT
Barriers<br><br><u>TFA:</u><br>Opportunity cost<br>Burden<br>Ethicality<br>Affective attitude	What do you find difficult?<br>*If necessary ask:*<br>*Confidence of the PLT?*<br>*Your own confidence?*<br>*Rejection by students?*<br>*Lack of time/patient care more important?*<br>*Effort to familiarize yourself/find out information?*<br>*Feel comfortable with the task/fun?*<br>*Disadvantages for patient care?*<br>*For your own postgraduate education?*<br>*For the training of students?*	What makes it difficult to involve trainees in student supervision?<br>*If necessary ask:*<br>*Confidence of trainees?*<br>*Your own confidence that trainees can take this on?*<br>*Rejection by students?*<br>*Lack of time/patient care more important?*<br>*Effort to instruct trainees on this?*<br>*Disadvantages for patient care?*<br>*For further training for trainees?*<br>*For training students?*
Role of student support in postgraduate education and in the self-image of GPs<br><br><u>TFA:</u><br>Coherence	How do you view trainees teaching students?<br>How important is it for you that you also have the opportunity to supervise students during your postgraduate education?<br>Would you like to supervise/teach students later as a GP?<br>To what extent is medical training part of the GP's duties?	Do you think it is right that trainees should teach students?<br>How important do you think it is that you are also given the opportunity to supervise students during your postgraduate education?<br>To what extent is medical training part of the GP's duties?
Outlook/future perspectives<br><br><u>TFA:</u><br>Ethicality<br>Self-efficacy	How should trainees be included in medical education in the future?<br>What support options are needed to enable more involvement?<br>To what extent should teaching become a regular part of postgraduate medical education?	How should trainees be included in medical education in the future?<br>What support options are needed to enable more involvement?<br>Should education/teaching become a regular part of postgraduate medical education?
Conclusion	Is there anything else you would like to add? Have I forgotten to ask something?	Is there anything else you would like to add? Have I forgotten to ask something?
