# Supplementary material for: Student supervision by trainee doctors in GP teaching practices: Win-win situation or additional burden? An interview study on current practices and acceptance
Source: GMS J Med Educ. 2024 Sep 16;41(4):Doc46. doi: 10.3205/zma001701 (PMC11474648; doi:10.3205/zma001701)
Supplement: Category system and example codes [file JME-41-46-s-002.pdf]

Attachment 2: Category system and example codes

Categories and example codes	
**Role of the trainees in practice operation**	
Role of the trainees in practice operation	*Trainee 6: Hm, well, I basically worked like a normal member of the team, I had my own room and we had a list of patient appointments that we just worked through together. (Trainee 6)*
**Role of the trainee in student supervision**	
Extent of supervision by trainees	*Normally I like it when the postgrad education assistants join in. We have written kind of a curriculum, that basically they go through PLT 5*
Agreements and initiative	*There was actually no discussion about whether I would do it, mainly they said, here you can go to (…) and then there they were (…) but I would definitely have had the courage to say if I had been uncomfortable. Trainee 2*
Type of supervision and supervision	*that in very simple consultation situations I sometimes say, I'll introduce the student to the patient and they can have a first go and I'll join in Trainee 2*
**Self-efficacy**	
Dealing with uncertainty and gaps in knowledge	*And, no, I don't actually think it's bad to say, I need to check something quickly or I need to read up on that or I need my boss's opinion to eventually come up with the right treatment somehow Trainee 5*
Advantages through professional experience	*but compared to the students who hadn't done anything practical, apart from an internship, I still thought that I was miles ahead. Trainee 8*
Problems	*On the other hand, it throws up some, it brings up aspects where I think, "Hm, I should really know that, being so close to becoming a specialist. So initially it's a bit of an ambivalent thing Trainee 2*<br>*We have a trainee doctor with us who isn't so knowledgeable (...) and I sometimes worry that students might learn something wrong. PLT 5*
Didactic preparation	*I don't think we've ever really been prepared for this, no. I think it's kind of expected that we just take the student along and show them what our normal process is, yes. And I think it's a bit of an assumption that it this will run on its own Trainee 5*<br><br>*I do think that it (didactic workplace training) is beneficial. So I think structured feedback and things like that are helpful. Trainee 7*
**Perceived effectiveness**	
Accessibility of the trainees for students	*and I think that they were happy because of course in terms of the age difference, so because the age difference is smaller and you're not as reluctant to ask more questions, well at least that's how I would feel and that was the impression I had too Trainee 7*
Other competences and learning content for students	*and when the trainee is there, then I'd always say, it's better to do ultrasound with her, she's better at it, she can explain it much better, it's better to do it with her if you want to learn it. Or in terms of caring for children (the trainee) was great PLT 8*

Attachment 2 to Gehrke-Beck S, Sonntag U, Schubert T, Madzharov M, Huenges B. *Student supervision by trainee doctors in GP teaching practices: Win-win situation or additional burden? An interview study on current practices and acceptance.* GMS J Med Educ. 2024;41(4):Doc46. DOI: 10.3205/zma001701

Input from several trainers for students	*So, I think, technically (...) you can learn more from (the PLT) now and in my case, maybe other things too, but you can learn a lot more from the trainee, how do I feel at the beginning, in dealing with uncertainties (...) just for different things. So, I think you need both. Trainee 6*
Role model for students	*in this practice, when I was doing my internship there, there were 2 young female assistant physicians whom I really admired because I thought: wow, if I could - well, almost idealizing things a bit - I'd like to be like that too or I'd like to be able to do that when I'm a doctor Trainee 4*
Teaching promotes your own learning	*as well as, let's say, purely in terms of subject matter, it often becomes clear what you know and what you don't know. So when I try to explain something, I quickly notice that I always do it that way, but I can't really justify it, like I don't fully get it. Trainee 6*
**Emotional attitude**	
Joy of teaching	*I really enjoy doing it, I'm happy when I can pass something on Trainee 8*
Self-confidence through a head start in knowledge	*that the two of them were so happy that she taught them that. Well, she was so proud, and that's also a real benefit, right. PLT 8*
**Opportunity costs**	
Time spent on student supervision	*it might have been better if I had organized it with him beforehand, so that we, that I could have made a little space in the calendar for the intern. Trainee 1*
Strategy: Organize teaching	*So now another idea of how to make this easier for the students is perhaps to determine within the practice what they can do. When I had psychosomatic discussions where I didn't include them, they often sat at the front desk so to clearly define that during that time they might go with the doctor's assistant and look over their shoulder to see what they are doing. So I wasn't really in a position to instruct them what they should to do when they weren't with me Trainee 2*
Trainees have more time for teaching	*so because my boss never doubled the number of appointments for us - she is of course also asked to train me in my postgraduate medical education rather than go through twice the number of patients with me, there are also requirements - so our schedule was, let's say, full, but not like we were under constant stress. And so it was actually still easy to manage with the student Trainee 8*
Workload reduction for PLTs	*definitely and the lunch break is definitely longer, if they do that (student supervision), it reduces my workload, that's true. PLT 8*
Benefits for patient care	*that I was also grateful, again for the input or that I perhaps gave a small research assignment (...) and also just having another set of eyes and ears by your side. Trainee 4*
Time required for didactic workplace training	*I see in my own case that there isn't enough time just for specialization training alone Trainee 7*

Attachment 2 to Gehrke-Beck S, Sonntag U, Schubert T, Madzharov M, Huenges B. *Student supervision by trainee doctors in GP teaching practices: Win-win situation or additional burden? An interview study on current practices and acceptance.* GMS J Med Educ. 2024;41(4):Doc46. DOI: 10.3205/zma001701

**Stress**	
Meeting the expectations of students and patients	*so I see the onus on myself to an extent, to offer them an educational and varied stay with us. I also see myself as having a responsibility to the patient in that I am not just relying on a student, but that I make the consultation just as effective for the patient as if I were alone with them Trainee 2*
Revealing uncertainty to students and patients	*answering questions on the spot while the patient is sitting there, for example, is perhaps a challenge. Because you are basically trying to explain something in the presence of the patient, which in an ideal scenario both should know Trainee 5*<br>*#*
**Ethics**	
Patient consent to students being present	*particularly perhaps with psychiatric patients, that could, I have the feeling, I'm not sure to what extent they can really say, to what extent they have the necessary sovereignty rather than just saying it's OK, perhaps because they're ashamed to say no Trainee 9*
**Coherence**	
Near-peer teaching in one's own learning experience	*I think the main factor that I saw at the time is that you had this positive experience, that someone took me along, so to speak, and that I also had the feeling that I could pass this part on to someone else after only a few weeks Trainee 1*
Preparation for later teaching tasks	*Especially since those who have completed specialization will at some point be practice employees or doctors themselves and if they have already laid a foundation there, perhaps the teaching in the teaching practices might also get a little better. PLT 6*
Problems with compulsory postgraduate education content	*I think it's good from a technical point of view if the trainees do it, but I would (...) be against it being included in the Regulations for Postgraduate Medical Education. Because that would turn into a bottleneck. There are many practices that do postgraduate training but don't have any students and then the trainees would have a problem finding a practice to gain certification. PLT 2*
Professional profile - Teaching as one's personal lifelong learning	*If you look at teaching and instruction as an opportunity for growth and consolidating your own knowledge, I also think it's an important part of the identity of a GP Trainee 9*
Professional profile - Teaching as a justification for your own actions	*I think every doctor should be able to let others look over their shoulder and justify their actions and explain them in such a comprehensible way so that someone else can apply and implement them. So that's the kind of transparency that I would actually demand from everyone in our profession. Trainee 2*
Professional profile - Teaching as a means to promote young talent	*Of course, I think it is incredibly important to get people interested in general practice, especially because we have such a shortage. Trainee 7*
Teaching as an additional task for those interested	*Yes, I don't think that it's for everyone, because I think some people don't like teaching. And I don't think they should do it either Trainee 3*
Promoting teaching in the practice	*And that's the only way you can actually keep up with knowledge and be forced to keep looking. Yes, I would like that, I would prefer it, you could say, if you got study credit for it. PLT 1*
